# 412. Clinical and demographic features associated with beta-lactamase producing Enterobacterales in an academic medical center in Loma Linda, CA

**DOI:** 10.1093/ofid/ofac492.489

**Published:** 2022-12-15

**Authors:** Eugene W Liu, James Pappas

**Affiliations:** Loma Linda University, Loma Linda, California; Loma Linda University, Loma Linda, California

## Abstract

**Background:**

Emergence of antibiotic resistant bacteria is a threat to public health. The aim of this study is to identify clinical and demographic features associated with beta-lactamase producing Enterobacterales, specifically extended spectrum beta-lactamase producing (ESBL) and carbapenem-resistant Enterobacterales (CRE), in patients at Loma Linda University Health, an academic medical center in Loma Linda, California.

**Methods:**

This retrospective case control study compared patients with/without beta-lactamase producing Enterobacterales. Cases and controls were identified from microbiology records with Enterobacterales isolated between January 1, 2020, and December 31, 2021. Case subjects were individuals with ≥1 CRE isolate (confirmed by nuclear amplification testing, NAT) and control subjects were those with ≥1 isolate that were all non-CRE. We identified isolates of interest, defined as the first CRE isolate in cases and the first non-CRE isolate for controls. For each isolate of interest and corresponding specimen collection date, we extracted electronic medical record data including inpatient antibiotics received in the preceding 6 months, specimen type, patient sex and age. We performed similar analysis with ESBL-producing Enterobacterales confirmed by NAT.

**Results:**

On multiple logistic regression, age (OR, 1.02; 95%CI, 1.01-1.04), isolation of klebsiella (10.75; 4.59-25.21), providentia (20.86; 2.47-176.17), and serratia (8.01; 1.59-40.40), receipt of non-carbapenem beta-lactams (1.06; 1.03-1.10), carbapenems (1.19; 1.12-1.27), doxycycline (1.26; 1.04-1.52), and tetracycline (1.89; 1.08-3.29), were independently associated with increased odds of CRE infection. Similarly, gender (2.59; 2.07-3.24), age (1.01; 1.01-1.02), isolation of proteus (1.47; 1.01-2.13), receipt of non-carbapenem beta-lactams (1.08; 1.06-1.11), carbapenems (1.33; 1.23-1.44), and vancomycin (1.07; 1.03-1.12) were associated with increased odds of infection with ESBL-producing Enterobacterales.

**Conclusion:**

Age and receipt of beta-lactams (including carbapenems) were associated with odds of infection with both CRE and ESBL-producing Enterobacterales in an academic medical center in Southern California, highlighting the importance of antibiotic stewardship.

**Figure:**
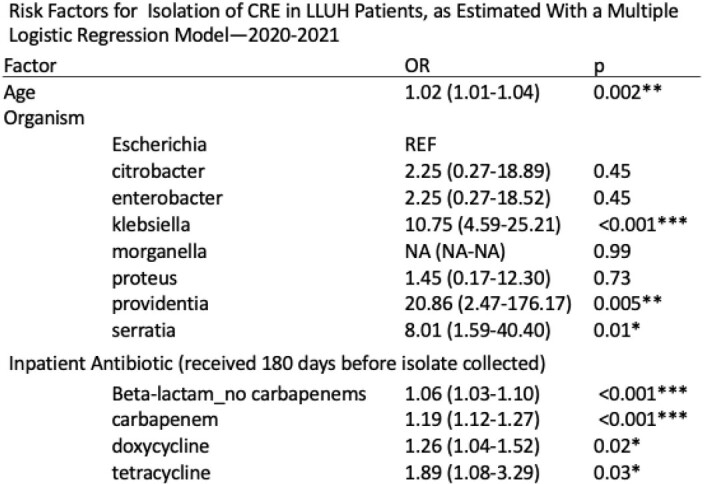

**Figure:**
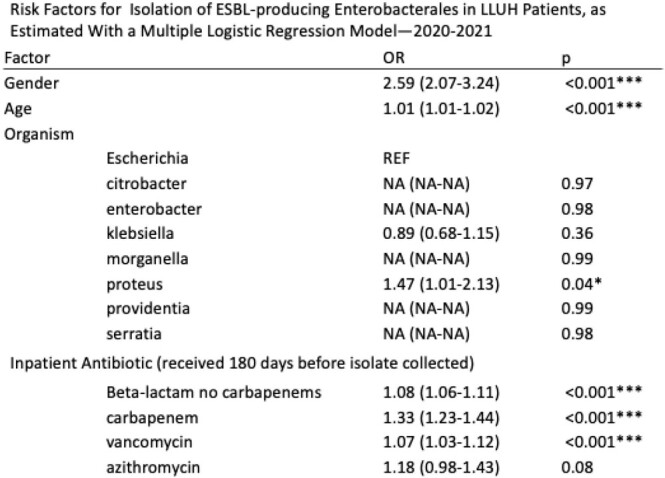

**Disclosures:**

**All Authors**: No reported disclosures.

